# Sensor-Based Technique for Manually Scanned Hand-Held Optical Coherence Tomography Imaging

**DOI:** 10.1155/2016/8154809

**Published:** 2016

**Authors:** Paritosh Pande, Guillermo L. Monroy, Ryan M. Nolan, Ryan L. Shelton, Stephen A. Boppart

**Affiliations:** 1 Beckman Institute for Advanced Science and Technology, University of Illinois at Urbana-Champaign, Urbana, IL 61801, USA; 2 Department of Bioengineering, University of Illinois at Urbana-Champaign, Urbana, IL 61801, USA; 3 Department of Electrical and Computer Engineering, University of Illinois at Urbana-Champaign, Urbana, IL 61801, USA; 4 Department of Internal Medicine, University of Illinois at Urbana-Champaign, Urbana, IL 61801, USA

## Abstract

Hand-held optical coherence tomography (OCT) imaging probes offer flexibility to image sites that are otherwise challenging to access. While the majority of hand-held imaging probes utilize galvanometer- or MEMS-scanning mirrors to transversely scan the imaging beam, these probes are commonly limited to lateral fields-of-view (FOV) of only a few millimeters. The use of a freehand manually scanned probe can significantly increase the lateral FOV. However, using the traditional fixed-rate triggering scheme for data acquisition in a manually scanned probe results in imaging artifacts due to variations in the scan velocity of the imaging probe. These artifacts result in a structurally inaccurate image of the sample. In this paper, we present a sensor-based manual scanning technique for OCT imaging, where real-time feedback from an optical motion sensor is used to trigger data acquisition. This technique is able to circumvent the problem of motion artifacts during manual scanning by adaptively altering the trigger rate based on the instantaneous scan velocity, enabling OCT imaging over a large lateral FOV. The feasibility of the proposed technique is demonstrated by imaging several biological and nonbiological samples.

## 1. Introduction

Optical coherence tomography (OCT), which utilizes low-coherence interferometry to perform optical ranging, is a noninvasive imaging technique capable of providing high resolution depth-resolved cross-sectional images of biological and nonbiological samples [1, 2]. Since its introduction in the early 1990s, OCT has found wide-spread applications in various areas ranging from biomedical imaging, where its diagnostic potential has been extensively studied in numerous medical and surgical specialities, such as ophthalmology [3–5], cardiology [6, 7], and oncology [8, 9], to nondestructive material evaluation and testing [10, 11]. In a traditional OCT system, a two-dimensional (2D) cross-sectional image, called a B-scan, is obtained by collating a sequence of successively acquired one-dimensional (1D) depth-resolved backscatter profiles, called A-lines, in a manner analogous to ultrasound imaging. In general, the A-lines are acquired at a constant rate while laterally scanning the imaging beam across the sample at a uniform velocity using either a galvanometric or microelectromechanical system (MEMS) scanning mirror. While widely used, galvanometer- and MEMS-based scanning have limited scan ranges or lateral fields-of-view (FOV) (several mm) making these systems unsuitable for applications where scanning over a larger range is desired.

Manually scanning or laterally moving a hand-held OCT imaging probe across tissue or a sample can be an alternative to galvanometer- or MEMS-based scanning. However, motion artifacts resulting from the nonuniform scan velocity of the probe can lead to a structurally inaccurate image of the sample. This problem has been recognized by several researchers, and a number of methods for overcoming this challenge have been proposed. These methods can be broadly classified as either sensorless [12–14] or sensor-based [15–17].

Sensorless methods exploit the correlation between adjacent A-lines to correct for motion artifacts. Since OCT data is inherently complex-valued, these methods are either intensity-based methods [12, 13], which use speckle decorrelation to track the probe motion, or phase-based methods [14], where the scan velocity of the probe is estimated based on the Doppler shift principle. While sensorless methods offer an inexpensive solution for correcting artifacts resulting from nonuniform scan velocity, their performance depends on the proper choice of algorithmic parameters, which in turn depends on the structure and scattering properties of the sample [12, 13]. Moreover, due to the computational expense involved, these methods are difficult to implement in real-time.

The basic idea of all sensor-based methods is to track the position of the imaging probe with respect to the sample. Among these methods, Ren et al. [15] proposed a method based on tracking the 3D position of four infrared (IR) LEDs (arranged in a tetrahedral fashion) attached to an OCT probe by recording a sequence of 2D images using a complementary metal oxide semiconductor (CMOS) camera. Two major limitations of this approach, which limit its applicability, are the requirement of a direct line-of-sight between the probe and the camera and a slow A-line acquisition rate limited by the frame rate of the camera. In another sensor-based approach, Yeo et al. [16] proposed a magnetic tracking method combined with signal processing algorithms for the reconstruction of freehand OCT scans. While this method does not suffer from the direct line-of-sight limitation of the previous method, it poses another challenge of reducing metal-induced magnetic field distortion. Moreover, the method requires careful sensor calibration for accurate tracking and postprocessing for overcoming the limited spatial resolution of the magnetic sensor. More recently, Iftimia et al. [17] have demonstrated a simple method for acquiring OCT images using a hand-scanning needle probe, which uses a linear encoder for sensing probe movement.

In this study, we present a sensor-based manual scanning technique for OCT imaging, where real-time feedback from an optical motion sensor is used to trigger the acquisition of A-lines. Since each A-line acquisition trigger corresponds to a fixed amount of relative displacement between the sensor and the tracking surface, the proposed technique is able to circumvent the problem of motion artifacts by adaptively altering the trigger rate based on the instantaneous scan velocity. The sensor used in this study is an inexpensive and small form-factor chip-on-board (COB) motion sensor most commonly used in laser-based computer mice. Our method is similar to that of Iftimia et al. [17] in that both methods utilize real-time feedback from a sensor to trigger A-line acquisition. However, unlike their method, where the probe movement is sensed relative to a fixed reference point on the encoder’s optical scale, which determines the scan range, our method detects probe motion by tracking the measured changes in position from sequential surface images recorded by the on-chip image acquisition system, which allows for truly freehand scanning.

## 2. Materials and Methods

In a traditional galvanometer- or MEMS-based scanning scheme, where A-lines are acquired at a constant temporal rate, two types of artifacts may arise from nonuniform scan velocity of the imaging probe. The first type, which we shall call the scaling artifact, results in regions imaged with a higher scan velocity to appear compressed in the acquired image, as compared to regions acquired with a lower scan velocity. The second type of artifact, called the smearing artifact, results from the intermittent pauses that occur during manual scanning. In this type of artifact, the region of pause appears as a smudge in the acquired image. This happens because, despite no relative movement between the sample and the probe, the A-lines are continuously being acquired at a predetermined constant rate. To overcome these motion artifacts, a mechanism is needed for triggering A-line acquisition such that triggers arrive only when the imaging probe moves by a fixed amount. To achieve this, we propose using a motion sensor to enable uniform spatial triggering of A-line acquisition as opposed to the uniform temporal triggering commonly used in the traditional galvanometer- and MEMS-based scanning OCT systems.

The sensing mechanism of the optical motion sensor used in this study is based on speckle tracking, in which, light from an IR laser is directed to the tracking surface, and the backscattered light from the surface, which forms a speckle pattern, is imaged onto a high-speed imaging sensor. By calculating the cross-correlation between the successive frames, the direction and magnitude of motion can be estimated. The proposed scanning technique is based around an integrated chip-on-board optical motion sensor (ADNS 9800, PixArt Imaging Inc.), which comprises a vertical-cavity surface-emitting laser (VCSEL) source, an imaging sensor, and a digital signal processor (DSP), which processes the stream of speckle images to determine the direction and distance of motion. The choice of the sensor was based on several desirable features including low-cost, high frame rate (up to 12,000 fps), high displacement resolution (15 μm), small form-factor, and low power architecture [18].

**Figure 1:**
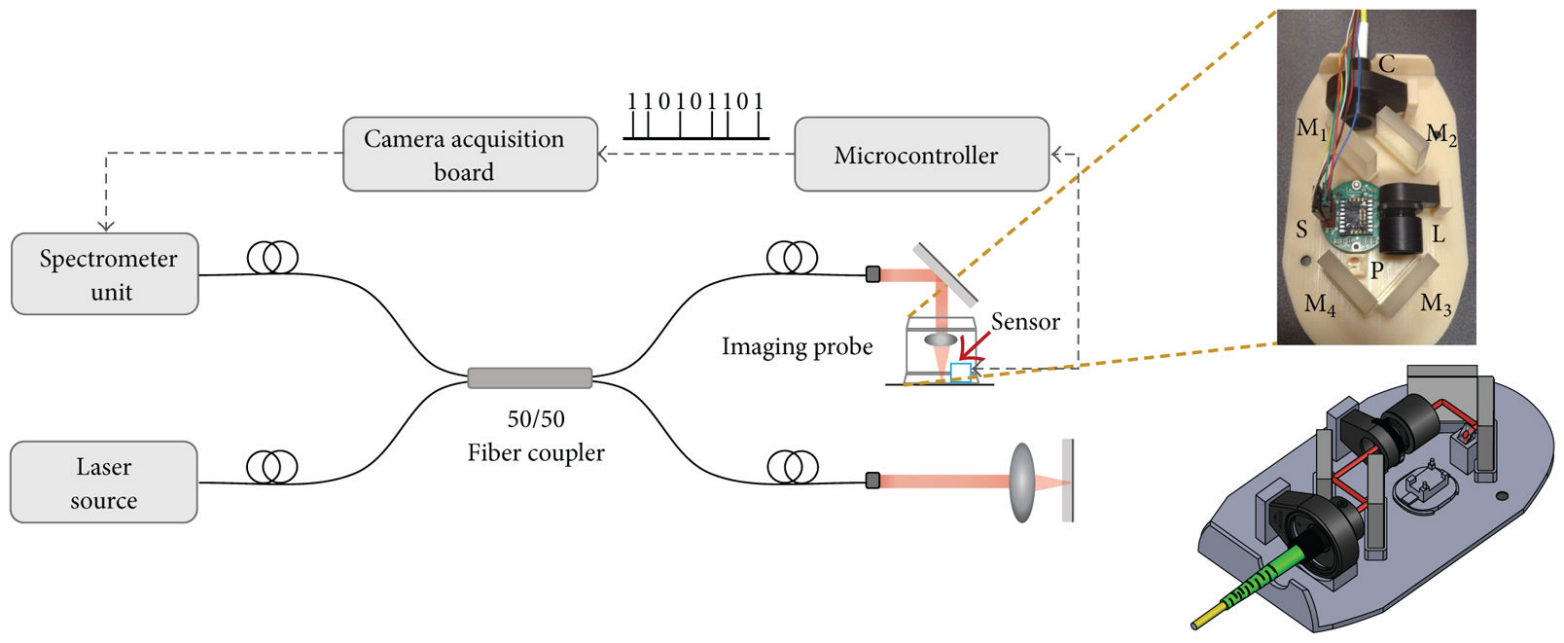
Schematic of the proposed imaging system. The hand-held imaging probe in the sample arm contains the motion sensor (cyan box) and the optics for focusing the OCT beam into the sample. The OCT beam path is shown in red in the SolidWorks rendering of the hand-held imaging probe. C: collimator, M_1_–M_4_: mirrors, L: focusing lens, P: right-angle prism, and S: motion sensor.

A basic schematic for OCT imaging using the proposed hand-held probe, sensor, and technique is shown in Figure 1. The imaging system is based around a Fourier-domain OCT system composed of a Michaelson interferometer with a broadband light source centered at 860 nm with a full-width-half-maximum (FWHM) bandwidth of 135 nm (T-860HP, Superlum), a spectrometer unit (Wasatch Photonics, UT) having a resolution of 0.04 nm, and a coupled 12-bit high-speed line-scan camera (spL4096-140km, Basler). The axial and transverse resolution of this OCT system were measured to be approximately 2.4 μm and 15μm in air, respectively. The imaging probe forms a part of the sample arm of the OCT system. As shown in Figure 1, light coming out from the fiber (shown as the red beam in the SolidWorks rendering) is delivered to the sample by means of collimation optics, mirrors, a focusing lens, and a right-angle prism. The motion sensor is attached to the base of the hand-held imaging probe (shown as cyan colored box in the schematic) and is interfaced with an external microcontroller (Arduino Uno R3; Atmega328) for reading out the motion parameters from the four-wire serial port of the sensor. The motion count signal (shown by a pulse train in Figure 1), which is available every time the sensor detects motion by an amount dictated by the resolution of the sensor, is used to trigger the acquisition of A-line.

## 3. Results and Discussions

**Figure 2:**
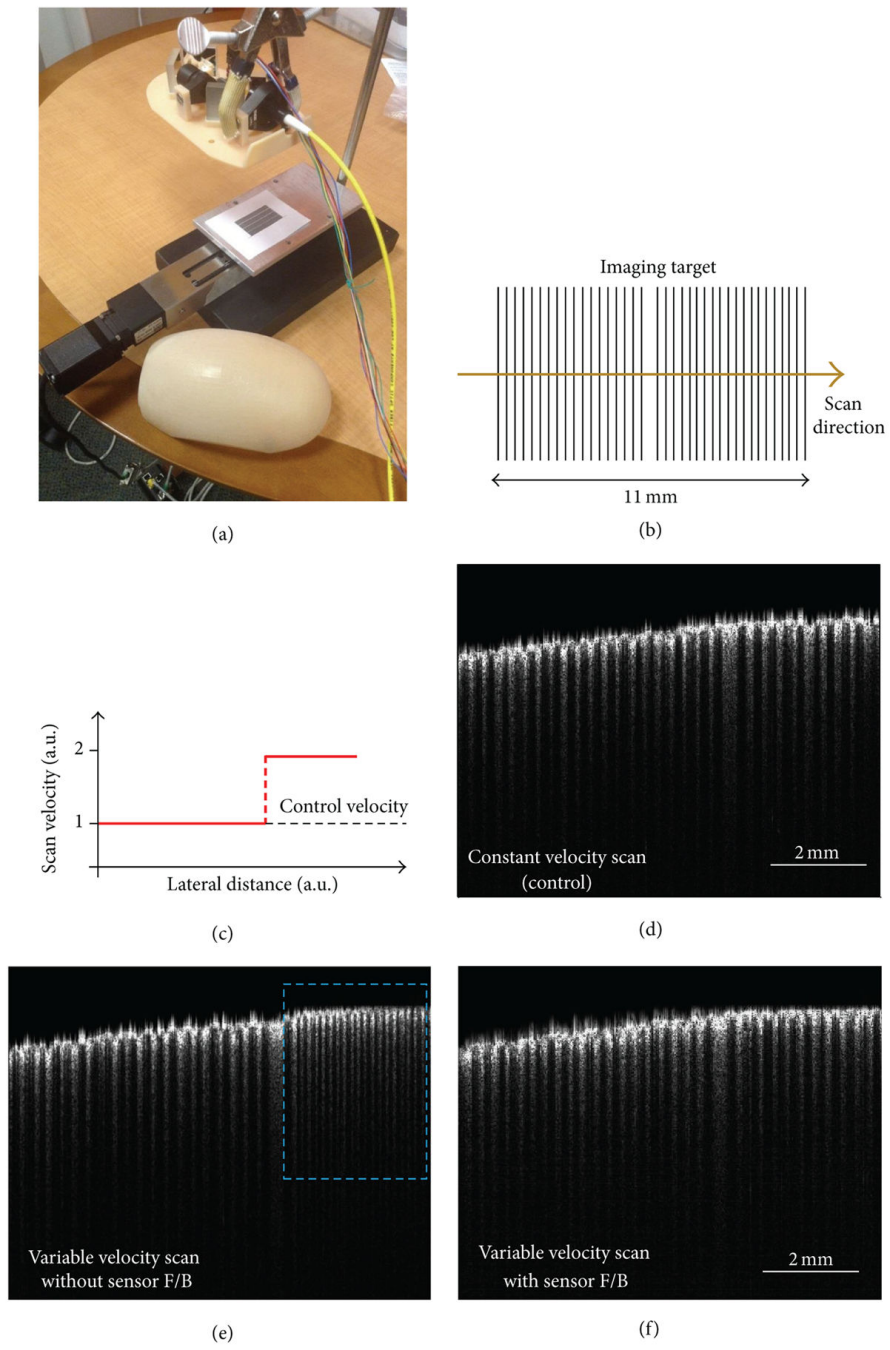
Imaging setup and results of testing the manual scanning technique. (a) Photograph of the setup used for testing the proposed technique showing the imaging probe mounted over a translation stage. (b) Pattern consisting of two sets of uniformly spaced bars printed on paper, used as the imaging target for testing the technique. (c)Nonuniform velocity profile fed to themotorized translation stage for simulating imaging artifacts in manual scanning. (d) Control B-scan obtained without sensor feedback while translating the sample at a constant speed shown as the black dashed line and labeled control velocity in (c). (e) B-scan obtained by acquiring A-lines at a constant rate (without sensor feedback) while translating the sample in accordance with the nonuniform velocity profile shown in (c). Scaling artifacts, marked by dashed cyan box, can be seen on the right side of the image. (f) B-scan acquired with feedback from the motion sensor while translating the sample in accordance with the nonuniform velocity profile shown in (c). Note the similarity with the control B-scan shown in (d).

Preliminary evaluation of the proposed technique was performed by imaging a printed pattern consisting of two sets of uniformly spaced bars as shown in Figure 2(b). To simulate nonuniform scanning velocity, the imaging target was moved under the imaging probe in a controlled manner by using a motorized translation stage. The probe itself was held fixed using a standard clamp stand, as shown in the photograph in Figure 2(a). To demonstrate the ability of the proposed technique to correct for the imaging artifacts resulting from nonuniform scanning velocity, images were acquired with and without sensor feedback, while moving the imaging target in accordance with the nonuniform velocity profile shown in Figure 2(c). For the case when the image was acquired in the absence of sensor feedback, A-line acquisition was triggered at a constant rate, as in the case of galvanometer- and MEMS-based scanning. Moreover, for comparison, a control image was also acquired without sensor feedback, while moving the imaging target at a constant velocity.

The imaging results are shown in Figures 2(d)–2(f). Figure 2(d) is the control image, where the inked and noninked regions of the printed pattern appear as a set of uniformly spaced dark and bright bands. Figure 2(e) was obtained without sensor feedback, while moving the imaging target at a variable velocity. As expected, the scaling artifact is clearly visible in the image, where the region on the right side, which was scanned with a higher velocity, appears compressed, as compared to the region on the left. More precisely, the second set of bars in the uncorrected image, which were scanned with a velocity two times the control velocity, have twice as many bars over the same lateral scan range as in the control image. It must also be pointed out that due to the nonuniform scanning velocity the lateral dimension (scan range) in the B-scan shown in Figure 2(e) was slightly larger than that of the control scan. Finally, the image shown in Figure 2(f) was obtained when feedback from the sensor was used to trigger the A-line acquisition. As can be seen in this case, the scaling artifact is no longer present and the image more closely matches the control image.

**Figure 3:**
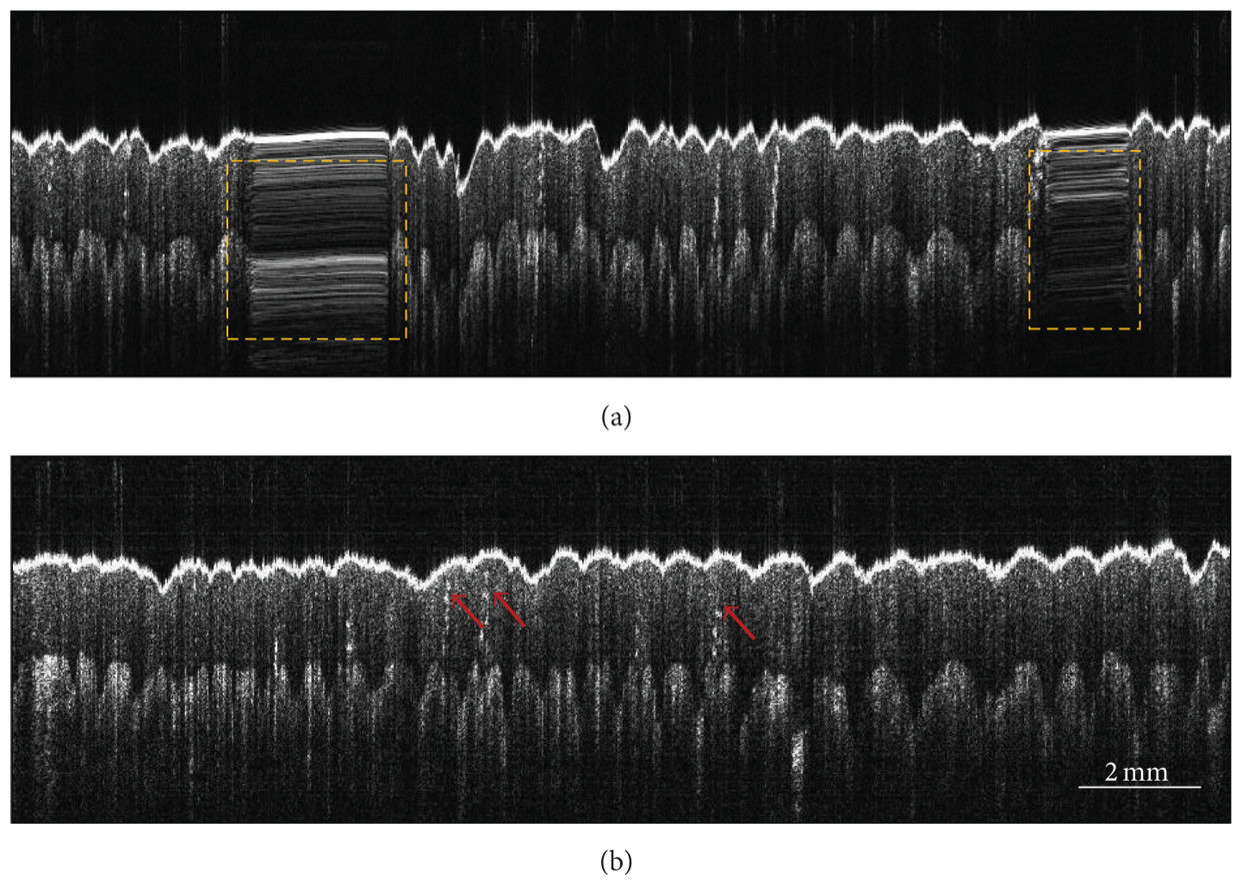
OCT B-scans of *in vivo* human palm skin acquired using the manually scanned imaging probe. (a) B-scan obtained without sensor feedback. Smearing artifacts are marked by dashed yellow rectangles. (b) B-scan obtained with sensor feedback showing the absence of imaging artifacts. Red arrows point to sweat ducts.

After validating the proposed technique in a controlled setting, OCT imaging of several biological and nonbiological samples was performed by manually scanning the imaging probe. For each sample, two images, acquired with and without sensor feedback, were recorded. Figure 3 shows the results of *in vivo* imaging of palm skin from a healthy human volunteer. Figure 3(a) shows the image obtained without sensor feedback, where severe smearing artifacts (marked by yellow dashed boxes) are visible. These artifacts are absent in Figure 3(b), which was obtained with sensor feedback. The junction between the dermal and epidermal layers of the skin can be easily identified in the images. Additionally, several fine structures, such as sweat ducts (red arrows in Figure 3(b)), which are not visible in Figure 3(a), can be resolved in Figure 3(b). It must, however, be noted that the B-scan acquired by using the feedback from the motion sensor shows a slight loss in image quality when compared to the corresponding uncorrected B-scan. The difference in image quality results from the difference in the number of A-lines constituting the two B-scans. As stated earlier, the sensor-based A-line acquisition was triggered by TTL pulses from the sensor, which are generated every time the imaging probe moves by an amount equal to the displacement resolution of the sensor, which is equal to 15 μm. On the other hand, the A-line acquisition, without sensor feedback, was triggered at a constant temporal rate of 33kHz. Due to this difference in the triggering schemes, the B-scan obtained without using the feedback from the sensor had 40 times more A-lines than the corresponding B-scan that was obtained by using the sensor feedback, which explains the difference in image quality between the two images shown in Figure 3.

**Figure 4:**
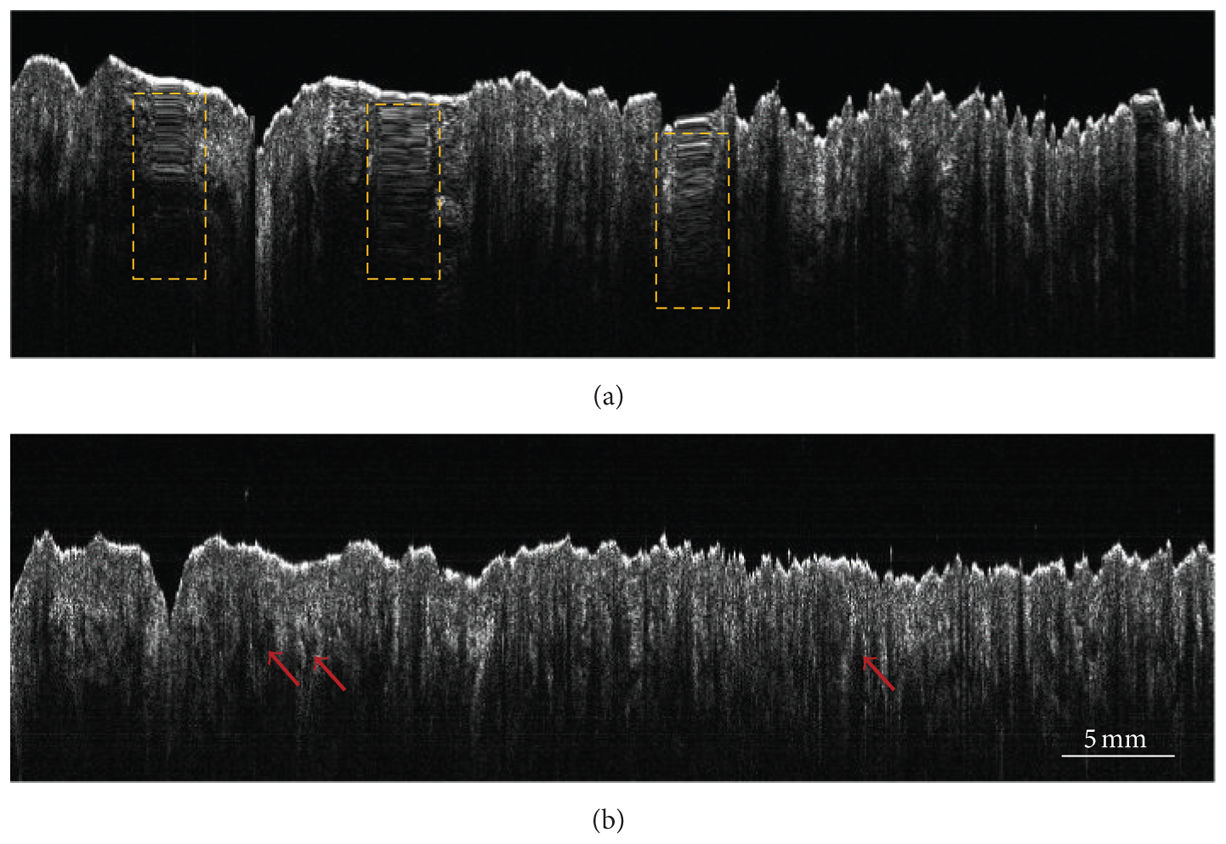
OCT B-scans of *in vivo* human cheek skin acquired using the manually scanned imaging probe. (a) B-scan obtained without sensor feedback. Smearing artifacts are marked by dashed yellow rectangles. (b) B-scan obtained with sensor feedback showing the absence of imaging artifacts. Red arrows indicate regions of low scattering, which correspond to sebaceous follicle infundibuli.

To demonstrate the advantage of the proposed technique for imaging sites that are otherwise difficult to image using a bench-top OCT system, OCT images of *in vivo* cheek skin from a healthy human volunteer were recorded. Just as in the previous case, Figure 4(a), which was obtained without sensor feedback, suffers from strong imaging artifacts. In comparison, the use of the sensor feedback resulted in a significantly improved OCT image, as shown in Figure 4(b). The dark regions, indicated by red arrows in Figure 4(b), identify the infundibuli, which are a part of sebaceous follicles and are known to be most dense in the cheek region of the facial skin [19]. It is also important to note that the lateral dimension of the images in Figure 4 is almost 5 cm, which is far greater than the typical scan range that can be achieved with a standard galvanometer- or MEMS-based scanning system.

**Figure 5:**
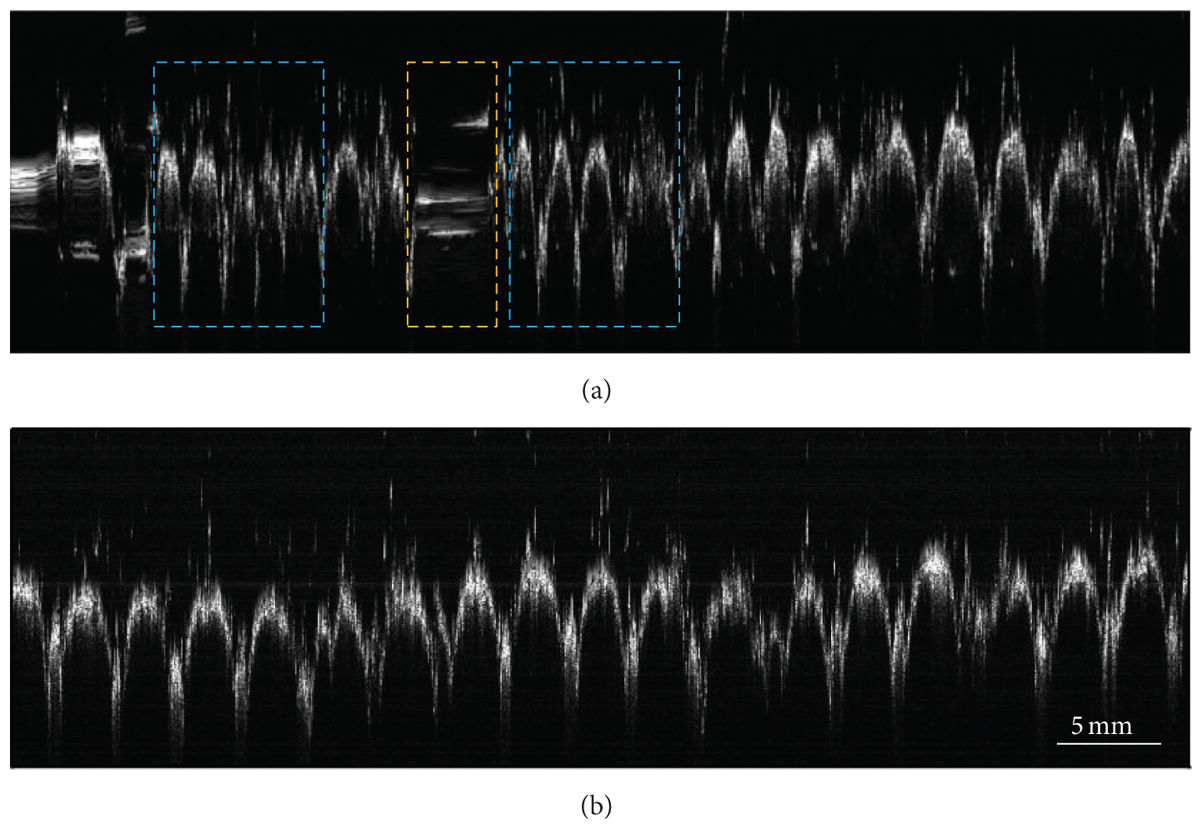
OCT B-scans of a denim fabric acquired using the manually scanned imaging probe. (a) B-scan obtained without sensor feedback. Scaling and smearing artifacts, marked by dashed cyan and yellow boxes, respectively, are clearly visible. (b) B-scan obtained with sensor feedback showing uniformly spaced peaks and valleys corresponding to standard periodic weave pattern.

Finally, as an application of our technique for material inspection, we imaged the standard twill weave pattern of a denim fabric sample. Figure 5(a) shows the image that was obtained without sensor feedback, where both scaling and smearing artifacts (marked by dashed cyan and yellow boxes, resp.) are clearly discernible. Figure 5(b), which was acquired using sensor feedback, shows uniformly spaced structures corresponding to the yarn, which constitutes the regular weave pattern. The number of weft yarns (valleys in the image) per centimeter estimated from the image was 3.8 yarns/cm, which was close to the physically measured value of 4.0 yarns/cm.

The theoretical maximum scanning speed, determined by the serial communication protocol of the sensor chip, was estimated to be approximately 2.5 cm/s, which is quite satisfactory for most freehand scanning applications. The practical use of this technique requires that the distance between the sensor and the tracking surface remains fixed. This is because the manufacturer-supplied lens assembly that is used with the sensor has a very small depth-of-field (DOF), more precisely, a minimum DOF of ±0.22mm above and below the focal plane [18]. While the problem of maintaining constant distance between the probe and the scanning distance can be mitigated, if not completely eliminated, for a contact-based imaging probe, as in the case of this study, it is of significant concern for noncontact imaging applications. A possible solution to this problem could be to replace the premanufactured lens assembly with custom-designed optics to achieve an increased DOF, which will be investigated in future studies. Another potential source of error that could affect the performance of the proposed technique is the angular tilt of the probe, which may produce false triggers. This problem was ameliorated in our design by using a wide bottomed base for the imaging probe and ensuring contact with the sample during imaging. Additionally, since the motion sensor is capable of detecting and reporting movement along both *x* and *y* directions, the proposed technique could be extended for 2D lateral scanning to acquire 3D OCT data.

## 4. Conclusion

In conclusion, we have presented a sensor-based manual scanning technique for OCT imaging. The proposed technique is able to overcome the problem of motion artifacts resulting from variations in scan velocity by adaptively altering the A-line acquisition trigger rate based on the instantaneous scan velocity. We demonstrated the feasibility of this scanning technique by imaging both biological and nonbiological samples over large scan ranges or lateral FOV that far exceed the capabilities of traditionally used galvanometer- or MEMS-based scanning techniques. While we discussed the scanning technique in the context of OCT, this technique and methodology could be used with any optical imaging modality where imaging over large lateral FOV is desired.
